# Targeting Mitochondrial Therapy in the Regulation of HPV Infection and HPV-Related Cancers

**DOI:** 10.3390/pathogens12030402

**Published:** 2023-03-02

**Authors:** Alfredo Cruz-Gregorio, Ana Karina Aranda-Rivera, Giovanni N. Roviello, José Pedraza-Chaverri

**Affiliations:** 1 Department of Cardiovascular Biomedicine, Ignacio Chávez National Institute of Cardiology, Juan Badiano No. 1, Colonia Section XVI, Tlalpan, Mexico City 14080, Mexico; 2 Laboratory F-315, Department of Biology, Faculty of Chemistry, National Autonomous University of Mexico, Mexico City 04510, Mexico; 3 Institute of Biostructures and Bioimaging, Italian National Council for Research (IBB-CNR), Area di Ricerca site and Headquarters, Via Pietro Castellino 111, 80131 Naples, Italy

**Keywords:** HPV infection, HPV-related cancer, mitochondria, oxidative stress, mitochondria therapy, compounds that target mitochondria

## Abstract

It has been previously proposed that some types of cancer cells reprogram their metabolic pathways, favoring the metabolism of glucose by aerobic glycolysis (Warburg effect) instead of oxidative phosphorylation, mainly because the mitochondria of these cells are damaged, thus displaying mitochondrial dysfunction. However, in several cancers, the mitochondria do not exhibit any dysfunction and are also necessary for the tumor’s growth and maintenance. Remarkably, if the mitochondria are dysfunctional, specific processes associated with the release of cytochrome c (cyt c), such as apoptosis, are significantly impaired. In these cases, cellular biotherapies such as mitochondrial transplantation could restore the intrinsic apoptotic processes necessary for the elimination of cancers. On the other hand, if the mitochondria are in good shape, drugs that target the mitochondria are a valid option for treating the related cancers. Famously, the mitochondria are targeted by the human papillomavirus (HPV), and HPV-related cancers depend on the host’s mitochondria for their development and progression. On the other hand, the mitochondria are also important during treatment, such as chemotherapy, since they are key organelles for the increase in reactive oxygen species (ROS), which significantly increases cell death due to the presence of oxidative stress (OS). In this way, the mitochondria in HPV infection and in the development of HPV-related cancer could be targeted to reduce or eliminate HPV infections or HPV-related cancers. To our knowledge, there was no previous review specifically focusing on this topic, so this work aimed to summarize for the first time the potential use of mitochondria-targeting drugs, providing molecular insights on the main therapeutics developed so far in HPV infection and HPV-related cancer. Thus, we reviewed the mechanisms associated with HPV-related cancers, with their early proteins and mitochondrial apoptosis specifically induced by different compounds or drugs, in which these molecules induce the production of ROS, the activation of proapoptotic proteins, the deactivation of antiapoptotic proteins, the loss of mitochondrial membrane potential (Δψm), cyt c release, and the activation of caspases, which are all events which lead to the activation of mitochondrial apoptosis pathways. This makes these compounds and drugs potential anticancer therapeutics that target the mitochondria and could be exploited in future biomedical strategies.

## 1. Introduction

**Figure 1:**
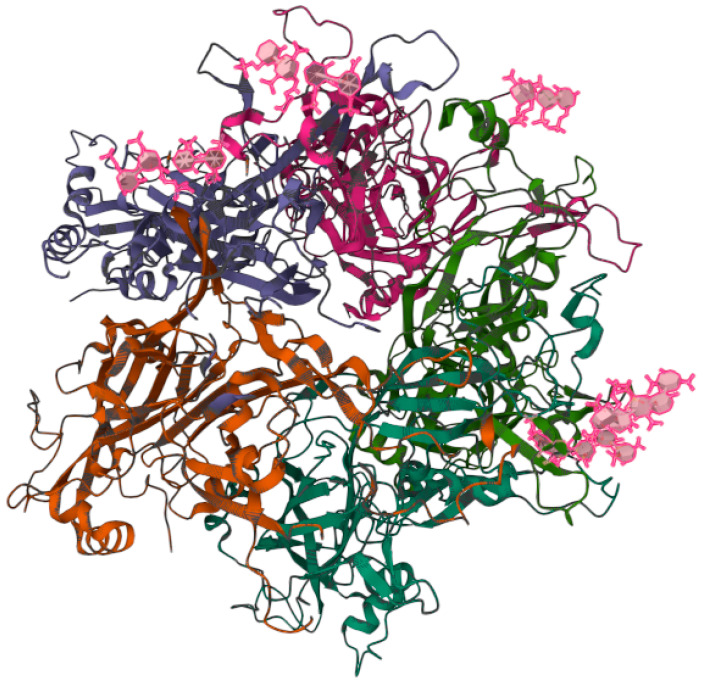
Three-dimensional structure of the heparin-bound pentamer of the L1 protein from human papillomavirus (HPV) Type 16. The image is publicly accessible at the link https://www.rcsb.org/3d-view/5W1O/1 (accessed on 6 February 2023) and corresponds to the structure with PDB ID 5W1O [10]. The viral capsid forms electrostatic and polar interactions with the anionic heparin fragments (pink) through the basic Lys-54, Lys-59, Lys-278, Lys-356, and Lys-361 and the polar residues Asn-57, Gln-194, and Thr-358. The interactions between HPV and the heparan sulfate oligosaccharide are of crucial importance, as they initiate the infection [10].

According to the World Health Organization (WHO), cervical cancer is the fourth leading cause of cancer death in women worldwide [1]. It has been established that persistent infection with high-risk human papillomavirus (HR-HPV) constitutes a key risk factor for the development of cervical cancer [2]. HR-HPV is also related to the induction of mucosal squamous epithelial malignancies of the penis, vulva, vagina, and oropharynx [3]. Although there are more than 200 types of HPVs, only HR-HPVs are able to induce cancer. The rest of the HPVs are low-risk HPV (LR-HPV), which are associated with skin warts and papillomatosis [4]. Among the HR-HPVs, viral types HR-HPV-16 and -18 are the most persistent [5]. These viruses are not enveloped viruses, with a capsid of approximately 55 nm [6]. The HPV capsid consists of the structural proteins L1 and L2 that house the viral genome [7], which is formed by a double-stranded circular deoxyribonucleic acid (DNA) of around 8,000 base pairs (bp) [8]. For study purposes, the HPV genome has been divided into three regions: (1) the early region (E: early) that encodes the genes involved in the replication of the viral genome and its maintenance (E1–E8); (2) the late region (L: late) that encodes the genes involved in the structural proteins of the capsid, namely L1 and L2; and (3) the long control region (LCR) that contains regulatory sites for transcription and replication of the HPV genome [9]. In cervical cancer, HPV’s life cycle begins when HPV infects the cells of the basal layer of the squamous epithelium of the cervix. HPV typically reaches these cells through wounds present in the epithelial layer, allowing the HPV L1 protein (Figure 1) to bind to the heparan sulfate proteoglycan receptors, which initiates the infection. It should be noted that the L1 of HR-HPV Type 16 forms a pentamer that is able to interact with the heparin oligosaccharides through the basic Lys-54, Lys-59, Lys-278, Lys-356, and Lys-361 and the polar residues Asn-57, Gln-194, and Thr-358 [10].

This interaction induces conformational changes in the viral capsid, allowing the L2 protein to interact with the host cell’s membrane [11,12]. Next, L2 promotes the internalization of HPV into the host cell. After internalization, HPV is transported through an early and a late endosome, where the capsid is disassembled during its transport to the Golgi network. Subsequently, the HPV genome enters the nucleus of the infected cell, where transcription and replication of HPV take place [8]. Both processes require basal epithelial cell differentiation because undifferentiated basal epithelial cells do not express key cellular transcription factors (TFs) such as specificity protein 1 (SP1) and transcription factor IID (TFIID), which are required to induce the expression of the early protein of HPV [13]. Thus, in the basal layer of the squamous epithelium, the viral transcription of E1, E2, E5, E6, and E7 and genome amplification are maintained at low levels [12]. As the epithelium is differentiated, the TFs that induce amplification of the viral genome are expressed, inducing HPV E1, E2, E5, E6, and E7 to increase their expression. Thus, in the middle layer, the E1 and E2 proteins of HPV augment their expression, increasing the replication of the HPV genome in high quantities. In turn, the E6 and E7 oncoproteins of HPV can interact with human telomerase reverse transcriptase (hTERT), p53, and retinoblastoma tumor suppressor (pRB), inducing cell immortalization, preventing cell death, and arresting the cell cycle [14,15]. E5 has been shown to disrupt the activation of the immune system, preventing the elimination of HR-HPV-infected cells [16]. In the upper layer, HPV E4 induces the disruption pf cell keratin, allowing the release of HPV virions after the L1 and L2 proteins house the HPV genome [8]. It should be noted that HPV virions are released until the epithelium is shed, since HPVs are nonlytic viruses [8]. HPV’s viral life cycle is completed over long periods of time during HPV infection without progressing to cancer; however, HR-HPV infections may progress to invasive cancers. A feature of these viruses that induces the progression to cancer is the integration of the viral genome of HR-HPV into the host cell’s genome. During this process, the expression of the E2 protein is lost, and since this is the negative regulator of the E6 and E7 oncoproteins, an overexpression of E6 and E7 occurs, causing genomic instability and cellular malignancy induced by the overexpression of these two oncoproteins [8]. It has been proposed that HPV-related cancers reprogram their metabolic pathways, favoring aerobic glycolysis (Warburg effect) [17]. However, the mitochondria are also necessary for the growth and maintenance of tumors. Thus, in HPV-related cancers, HR-HPV proteins interact with the mitochondria, modifying their function and influencing the lifecycle of HR-HPV and cell transformation [18]. Interestingly, LR-HPVs also influence the mitochondria. For example, Sun et al. [19] demonstrated that LR-HPV-6 E6 stabilizes p53 in the cytosol. This induces p53 to interact with the pro-apoptotic mitochondrial protein Bak, which undergoes a conformational change and oligomerization, allowing the release of pro-apoptotic factors and inducing mitochondrial apoptosis. Therefore, unlike the HR-HPV E6 protein, which can induce the degradation of p53 [20], LR-HPV E6 manages to stabilize this oncoprotein by inducing apoptosis in response to a stress stimulus such as exposure to UVB light [21]. This large difference between HR-HPV and LR-HPV is clearly associated with the induction of malignancy by HR-HPV E6 and the low potency of malignancy by LR-HPV, where, in cancer, HR-HPV E6 induces the degradation of p53, decreasing apoptosis even in response to any stress stimulus. The mitochondria are also important during cancer treatments, such as chemotherapy, since they are key organelles for the increase in reactive oxygen species (ROS), which significantly increase cell death due to the presence of oxidative stress (OS). In this way, HPV-related cancer proteins and the mitochondria in HPV infection and in the development of HPV-related cancer could be targeted to reduce or eliminate HPV infections or HPV-related cancers through mitochondrial apoptosis. Here, we focus on the potential of mitochondria-targeting therapies and provide molecular insights on the main cures developed so far using this approach in HPV infections and HPV-related cancer. 

## 2. Mitochondria, Oxidative Stress (OS), Apoptosis, and the Development of HPV-Related Cancer 

The mitochondria play a fundamental role in the development of cancer, since they regulate metabolism, growth, survival, and cell apoptosis. In fact, the mitochondria coordinate processes such as oxidative phosphorylation (OXPHOS), the tricarboxylic acid cycle (TCA), the electron transport system (ETS), fatty acid oxidation (FAO), and the synthesis of aminoacids, lipids, and nucleotides [22]. Remarkably, during OXPHOS, electrons can leak, inducing the reduction of O_2_ to superoxide anion (O_2_^•−^), a ROS which triggers the production of other ROS species, such as hydrogen peroxide (H_2_O_2_) or the hydroxyl radical (^•^OH) [23]. Remarkably, ROS can diffuse into the cytosol and nucleus, damaging cytosolic and nuclear organelles, and ROS produced in the cytosol can even affect the mitochondria in a feedback loop between the cell organelles. Because of this, ROS must be removed, but not completely, by an antioxidant system that includes antioxidant enzymes such as superoxidase dismutase (SOD), glutathione peroxidase (GPx), or catalase (CAT), and non-enzymatic antioxidants such as glutathione (GSH) and vitamins [24,25]. The induction and activation of antioxidant enzymes and the enzymes associated with the production of GSH are induced by different transcriptional factors, such as nuclear factor erythroid 2-related factor 2 (Nrf2), which, in turn, are activated by the increase in ROS levels to reduce them, which allows redox homeostasis to be achieved [26,27,28]. Redox homeostasis is necessary because ROS species are used as secondary messengers, activating several signaling pathways [29]; however, at high concentrations, ROS can induce OS and oxidative cellular damage, such as damage to the DNA, which is closely related to mutagenesis and the development of cancer [30,31,32,33,34]. Oxidative DNA damage triggers the protein kinases ataxia-telangiectasia mutated/RAD3-related (ATM/ATR), leading the DNA repair machinery to activate the DNA damage response (DDR) signaling pathway [35]. The DDR signaling pathway activates the tumor suppressor p53, arresting the growth and the cell cycle, which permits the repair of DNA damage [36,37]. If the DNA is not repaired, p53 induces apoptosis, eliminating potentially cancerous cells [38]. However, in HR-HPV infections, E6 induces the degradation of p53 via the proteasome, which promotes the accumulation of DNA damage, prevents death by apoptosis, and thus increases the risk of cancer developing [20]. 

It should be noted that p53 induces apoptosis as a response to cellular stress, mitochondrial dysfunction, and stimuli such as DNA damage in a process known as mitochondrial apoptosis or intrinsic apoptosis, since key mitochondrial proteins are released from the mitochondria to the cytosol [39]. In other words, in mitochondrial apoptosis, p53 activates proapoptotic proteins, such as the BH3-only family proteins (Bad, Bim, and p53 upregulated modulator of apoptosis (PUMA), to cite only a few) and the Bcl-2 family proteins (Bcl-2 homologous antagonist/killer (Bak), Bcl-2-associated X protein (Bax), and Bok), as well as deactivates antiapoptotic proteins such as Bcl-2 and Bcl-XL. Proapoptotic proteins promote the release of cyt c, which starts mitochondrial apoptosis, binding to apoptotic protease activating factor-1 (Apaf1) to form the apoptosome [40]. The apoptosome activates caspase-9 [41,42], which, in turn, activates caspase-3 [43], promoting the disassembly of the cytoskeleton and the disintegration of the apoptotic bodies and even inducing the activation of endonuclease caspase-activated DNase (CAD). CAD, in turn, cuts chromosomal DNA, forming DNA fragments [44]. Apoptotic cells express phosphatidylserine on their surface in order to be recognized and phagocytized by non-inflammatory phagocytic cells [45]. As mentioned above, during HR-HPV infections and in HPV-related cancer, p53 is downregulated due to the overexpression of the E6 oncoprotein, causing intrinsic apoptosis to be absent, inducing a predisposition to the development of cancer [20]. In the next section, we describe the main relationships among HR-HPV proteins, mitochondria, OS, and apoptosis.

## 3. HR-HPV Proteins, Mitochondria, OS, and Apoptosis

**Figure 2:**
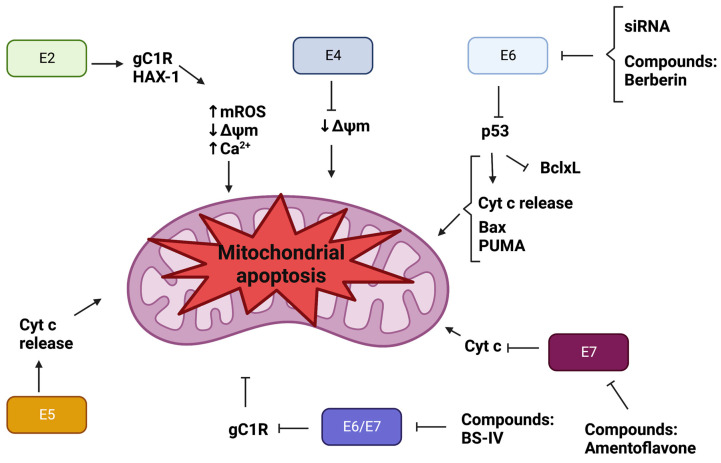
Human papillomavirus (HPV) proteins and compounds induce mitochondrial apoptosis in HPV-related cancers. HPV proteins such as E2, E4, and E5 can bind to the mitochondria, inducing mitochondrial apoptosis. On the other hand, E6 and E7 oncoproteins protect against mitochondrial apoptosis; however, different compounds, such as berberine, reduce the levels of these oncoproteins, reactivating p53, which activates proapoptotic proteins such as Bcl-2-associated X protein (Bax) and the p53 upregulated modulator of apoptosis (PUMA), and deactivates antiapoptotic proteins such as Bcl-xL, reducing the mitochondrial membrane’s potential (Δψm) and promoting the release of cytochrome c (cyt c). The latter induces the activation of caspase-9 and -3, resulting in mitochondrial apoptosis.

Although the major function of the E2 protein of HPV is to allow the interaction of E1 with the HPV genome, E2 also suppresses cell growth and induces senescence caused, in part, by a repressed expression of E6 and E7, which induces the increase in p53 levels, and leads to cell cycle arrest [46]. Furthermore, it has been shown that the E2 of HR-HPV-18 interacts with different proteins from mitochondrial Complexes III and IV, and ATP synthase, which is related to the mitochondria’s loss of the cristae structure and the induction of the release of mitochondrial ROS (mROS) [47]. The E2 of HR-HPV-18 also increases ROS in whole C33A cells, resulting in a decrease in GSH levels and the induction of OS [48]. As mentioned above, ROS may act as a secondary messenger activating different key proteins in signaling pathways associated with cancer, one of which is the hypoxia-inducible factor 1α (HIF 1α). Lai et al. [47] showed that the release of mROS caused by the expression of HPV-18 E2 stabilizes HIF 1α, inducing the expression of its target genes for 3-phosphoinositide-dependent protein kinase 1 (PDK1), carbonic anhydrase IX (CAIX), and vascular endothelial growth factor (VEGF). Importantly, PDK1 inhibits the pyruvate dehydrogenase (PDH), the enzyme that promotes the conversion of pyruvate to acetyl-CoA, which is the main molecule that fuels the TCA in the production of nicotinamide adenine dinucleotide (NADH), which, in turn, is used as a reducing agent during OXPHOS [47]. Thus, the increase in PDK1 is associated with the reduction in OXPHOS, inducing an aerobic glycolysis profile. In aerobic glycolysis, the acidification of the cell increases, so the cells need to reduce this effect. A protein involved in this deacidification is CAIX, which is stimulated by HIF 1α, previously activated by E2 [47]. Thus, HPV-18 E2 prevents OXPHOS by associating with key mitochondrial proteins such as complex III and IV and ATP synthase, inducing the release of mROS, the stabilization of HIF-1α, PDK1, and the upregulation of CAIX, resulting in the induction of aerobic glycolysis [47]. Gao et al. [49] found that E2 from HR-HPV-16 also interacts with the mitochondria, inducing inflammation and the immune response to infection. This interaction also induces mitochondrial dysfunction, as well as the production of mROS, and leads to cell death via apoptosis. Overexpression of E2 induces the expression of the receptor gC1qR, which mediates growth arrest, morphological abnormalities, and apoptosis inside the mitochondria. Therefore, when the E2 of HR-HPV-16 induces the expression of gC1qR, mitochondrial dysfunction and apoptosis are induced. The increase in ROS generation and cytosolic Ca^2+^ levels, plus a decrease in mitochondrial membrane potential (Δψm), and the loss of the mitochondrial morphology characterize mitochondrial dysfunction (Figure 2) [50]. In line with the latter observation, it has been shown that E2 from HR-HPV-16 decreases the expression of hematopoietic cell-specific protein 1-related protein X-1 (HAX-1). This mitochondrial membrane protein induces cell growth, invasion, and metastasis in cervical cancer. Thus, when E2 is expressed, the levels of HAX-1 decrease, inducing an increase in the generation of ROS but also a decrease in ΔΨm, alongside the loss of the mitochondrial morphology, promoting mitochondrial apoptosis overall [51].

As shown above, the E4 protein from HR-HPV-16 binds to the cytokeratin network, provoking its collapse in order to induce the release of HR-HPV particles [52]. However, this early protein also binds to the mitochondria. This interaction induces the mitochondria to detach from the cytoskeleton microtubules, reducing the ΔΨm and promoting apoptosis [53]. Unfortunately, to the best of our knowledge, this is the only investigation conducted so far on the effect of E4 on the mitochondria, a theme that deserves further research because E4 is part of a key process during the lifecycle of HPV, namely the release of HPV virions. 

As for the E6 of HR-HPV, it has been shown that E6 oncoproteins from HR-HPV-16 and -18 enhance mitochondrial metabolism in a head and neck squamous cell carcinoma (HNSCC) cell model, increasing cell respiration and the expression of mitochondrial complexes I–IV and ATP synthase. Interestingly, E6s do not promote the production of ATP but increase the leakage of mitochondrial respiration parameters, augmenting the production of ROS, which ultimately results in DNA damage [54]. The E6 genes of HR-HPV can also be processed through the cell splicing process, during which spliced products known as E6* are formed [55]. The isoform HR-HPV-16 E6* also induces mitochondrial dysfunction, the production of ROS, and OS in cervical cancer cells [56]. Although E6 promotes mitochondrial dysfunction, OS, and DNA damage, it does not induce apoptosis, primarily because E6 decreases p53 levels. This effect also was shown in primary epidermal keratinocytes exposed to ultraviolet B (UVB) radiation, where the expression of E6 from HR-HPV-18 avoided the release of cyt c and mitochondrial apoptosis [21]. However, the downregulation of E6 would restore p53 levels, inducing apoptosis. In this respect, the silencing of E6 from HR-HPV in CaSki cells led to an increase in p53 [57], which led to mitochondrial apoptosis induced by the release of cyt c [58]. Consistent with the latter finding, the blockage of E6 by siRNA in HeLa cells stimulated the transcription of the PUMA promoter. This led to the translocation of Bax to the outer mitochondrial membrane, opening the pores for releasing of cyt c, which activated caspase-3 to trigger apoptosis. Additionally, the inhibition of Bax reverted apoptosis, indicating that E6 requires the blockage of Bax to avoid apoptosis [59]. Thus, E6 prevents mitochondrial apoptosis, which could be induced by the downregulation of E6 by direct silencing of the E6 gene. Interestingly, different compounds such as luteolin, CAF24, GRIM-19, and quercetin may disrupt the interaction between E6 and E6AP (E6-associated protein), an interaction that permits the binding with p53 and, in consequence, its degradation via the proteasome is avoided, which stabilizes p53, promoting apoptosis in HPV-positive cell lines [60,61,62]. E6 is also sensitive to OS-derived ROS inducers such as H_2_O_2_. Hence, in human cervical cancer cells, H_2_O_2_ leads to apoptosis by inducing the phosphorylation of Bax via the extracellular signal-regulated kinase (ERK) pathway. It was shown that treating SiHa and CaSki cells with H_2_O_2_ induces the phosphorylation and activation of ERK and c-Jun N-terminal kinases (JNK), which are proteins involved in apoptosis [63]. The activation of both proteins promotes the phosphorylation of Bax, inducing its translocation to the outer mitochondrial membrane. Consequently, cyt c is released and apoptosis is induced. H_2_O_2_ also activates p73, a member of the p53 family, which inhibits the antiapoptotic protein Bcl-XL and stimulates apoptosis in SiHa and CaSki cells [63]. Thus, cervical cancer cell lines expressing E6 are more sensitive to the overproduction of ROS. 

The E7 oncoprotein of HR-HPV binds to and degrades several cellular proteins, such as the pRB proteins, which contribute to cellular transformation [64]. Moreover, it has been shown that E7 protects against OS agents such as H_2_O_2_, which prevents the death of cancer cells [65]. Regarding the mitochondria, it has been shown in human keratinocytes expressing the E7 oncoprotein of HR-HPV-16 that histone deacetylase (HDAC) inhibitors induce mitochondrial apoptosis [66]. Furthermore, amentoflavone, an active phenolic compound isolated from *Selaginella tamariscina*, decreases the expression of E7 oncoprotein in SiHa and CaSki cervical cancer cells, inducing the release of cyt c and the activation of caspase-9 and -3 to trigger mitochondrial apoptosis [67]. Remarkably, E6 and E7 oncoproteins are expressed by bicistronic mRNA, and thus both oncoproteins are expressed simultaneously in HPV-related cancers. It has been shown that E6/E7 oncoproteins inhibited the expression of gC1qR, preventing mitochondrial dysfunction, the activation of caspase-3, and mitochondrial apoptosis [68]. However, anti-inflammatories such as buddlejasaponin IV (BS-IV) are able to induce apoptosis via the mitochondria-dependent pathway, blocking the progression of HPV-induced oral carcinogenesis caused by E6/E7 oncoproteins [69]. Lipids such as docosahexaenoic acid (DHA) also induces the degradation of E6/E7 oncoproteins through the activation of the cellular ubiquitin–proteasome system (UPS), inducing the production of mitochondrial ROS and mitochondrial apoptosis [70]. Therefore, the protection against mitochondrial apoptosis obtained by the expression of E6, E7, or E6/E7 is overcome by different compounds such as DHA, which induce a decrease in E6/E7, inducing apoptosis. This makes them good candidates as anticancer compounds in HPV-positive cancers.

## 4. HPV Proteins Sensitize Cancer Cell Death through Mitochondrial Apoptosis or Mitophagy

Although HPV proteins prevent cell death, it has been shown that some HPV proteins also induce apoptosis via a mitochondria-dependent pathway. For example, the E5 oncoprotein of HR-HPV-16 was shown to sensitize HaCaT cell lines treated with a hyperosmotic concentration of sorbitol, inducing the release of cyt c; the activation of caspase-3, -8, and -9; and cleavage of poly-(ADP-ribose) polymerase (PARP), demonstrating the activation of mitochondrial apoptosis [71]. As for the E6 oncoprotein of HR-HPV-16, it was shown that this oncoprotein sensitizes the cervical cancer cell lines CaSki, HeLa, and SiHa to atractyloside, an inducer of mitochondrial outer membrane permeabilization (MOMP), inducing cell death via apoptosis [72]. This sensibilization was also induced by other biomolecules, such as tumor necrosis factor (TNF), but in an ovarian and a colon cell model. In this model, E6 from HR-HPV-16 increases susceptibility to the mitochondrial apoptosis induced by TNF, which inhibits nuclear factor kappa light-chain-enhancer of activated B cells (NFκB), promoting the release of cyt c and the activation of apoptosis [73]. On the other hand, it has been reported that E7 is associated with pRB, inducing the release of E2F5. E2F5 binds and stabilizes dynamin-related protein 1 (Drp1), an upstream inducer of lethal mitophagy, promoting cell death and tumor suppression in HPV(+) HNSCC both in vitro (cell culture) and in vivo (xenotransplant in mice) [74]. Thus, the activation of Drp1 via the expression of the E7 oncoprotein induces lethal mitophagy, providing a potential opportunity for an anticancer strategy. This is related to the evidence that HNSCC HPV(+) cancers are closely related to the mitochondrial metabolism, and this feature could be associated with a better response to treatment, giving it an advantage over other HPV-related cancers, such as cervical cancer, which are more associated with the aerobic glycolysis metabolism [17,25,54]. Although several examples in the literature [25,54] have shown a tendency towards inducing the death of cancer cells by HPV proteins, more research is needed to investigate these effects since they could be dependent on the concentration of the HPV proteins or on the model used for the study. Therefore, in-depth examinations of these effects could reveal new mechanisms for the reduction or eradication of HPV-associated cancers.

## 5. Mitochondrial Therapy in HPV-Related Cancers

**Figure 3:**
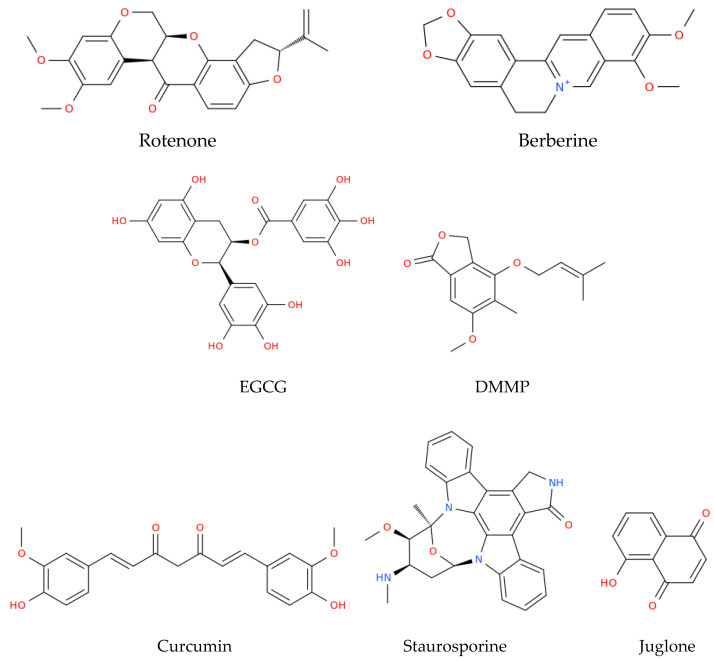
Structural representation of some of the main natural compounds that are able to target the mitochondria, as described in this review. EGCG, epigallocatechin gallate; DMMP, 4-(3′,3′-dimethylallyloxy)-5-methyl-6-methoxy-phthalide.

HPV-related cancers display several degrees of alteration in the mitochondrial metabolism; however, all are dependent on the metabolism of the mitochondria, both for completing their energy metabolism and for avoiding cell death [54,75,76,77]. Because of these roles, the mitochondria are an attractive target for HPV-related cancer therapy, even at early stages such as during HPV infection. For instance, Zhain et al. [78] have shown that alterations in the mitochondrial DNA (mtDNA) are present in the framework of HPV infection. This group reported that the C150T polymorphism present in the mtDNA D-loop was related to HPV infection in patients with cervical cancer. Thus, people that have this polymorphism are more prone to HPV infections. Finding this polymorphism in the population gives an advantage against possible HPV infections, since people can be alerted about their propensity to be infected by HPV and develop HPV-related cancers. However, apart from diagnostics, the mitochondria may also be a therapeutic target in cancer. It was shown that in models of cancer such as HeLa, the development of cancer cell is highly glucose-dependent, and the use of rotenone (Figure 3), a strong inhibitor of Complex I ETS, induces the production of ROS, arrests growth, and leads to mitochondrial apoptosis [79]. Moreover, the cycle of HeLa cells was arrested at the G0/G1 phase, which was accompanied by the release of cyt c and the second activator of caspase (Smac)/direct IAP binding protein with low pI (DIABLO) from the mitochondria to the cytosol, triggering the activation of procaspase-9 and -3 and the induction of cleaved PARP under treatment with triphenyl tin (TPT)-benzimidazolethiol [80]. 

The latter compound was used to achieve the downregulation of E6 from HPV, which restored the expression of p53, inducing mitochondrial apoptosis [80] (Table 1). The cyano-derivative of 11-keto-β-boswellic acid, butyl 2-cyano-3, 11-dioxours-1,12-dien-24-oate (BCDD), also reduced the expression of E6 in the nucleus of HPV-18 HeLa cells, which promoted the accumulation of active p53 cells, followed by the inhibition of anti-apoptotic Bcl-2, the augmentation of Drp-1, and disruption of mitochondrial functions, ultimately causing mitochondrial apoptosis [81]. HeLa cells also underwent mitochondrial apoptosis when these cells were treated with berberine, a natural alkaloid derived from the medicinal plant *Berberis vulgaris*, which is endowed with different biological properties [82]. The treatment with berberine decreased the levels of E6 and E7, increasing the levels of p53 and pRB; this reduced the cells’ viability through the loss of ΔΨm, the activation of caspase-3, and the induction of cleaved PARP [83]. Moreover, in experiments conducted on HeLa cells, some polyphenols present in green and black tea, such as epigallocatechin gallate (EGCG) and theaflavins (TF), respectively, induced the production of ROS, the release of cyt c, and cleavage of caspase-9 and -3, triggering intrinsic apoptosis [84]. Moreover, 4-(3′,3′-dimethylallyloxy)-5-methyl-6-methoxy-phthalide (DMMP), an agent from the endophytic fungus *Pestalotiopsis photiniae,* also induced mitochondrial apoptosis in HeLa cells [85]. This potential antitumor compound arrested the cell cycle in the G1 phase, induced the loss of ΔΨm, enhanced p53 levels, and increased the mRNA expression of pro-apoptotic Bcl-2 family genes (PUMA, NOXA, Bax, Bad, and Bim) in order to promote mitochondrial apoptosis [85]. Curcumin, a phytopolylphenol isolated from *Curcuma longa*, also induced intrinsic apoptosis in cervical cancer cell lines through the release of cyt c and the activation of caspase-3 and -9 [86]. In line with the latter drug, some compounds, such as atovaquone, have also shown anticancer properties in cervical cancer cell lines and in vivo models. In particular, this antiprotozoal drug induced intrinsic apoptosis by inhibiting mitochondrial complex III both in vitro, in SiHa cells, and in vivo, in a cervical cancer xenograft mouse model [87]. On the other hand, the use of drugs such as staurosporine, a potent inhibitor of multiple protein kinases, decreased the expression of E6 and E7 oncoprotein in Caski and HeLa cervical tumor cells. This compound induced an increase in p53, the release of cyt c into the cytosol, and the activation of caspases-9 and -3, leading to PARP cleavage and apoptosis related to the mitochondria [88]. 

Juglone, an antioxidant extracted from the roots, leaves, nut hulls, wood, and bark of *Juglans mandshurica*, was able to decrease the levels of Bcl2 and increased Bax, causing the release of cyt c from the mitochondria, inducing mitochondrial apoptosis and cell cycle arrest at the G2/M phase in CaSki cell lines [89]. Similar effects were observed with a chloroform extract of Rasagenthi Mezhugu (RM), a formulation from traditional Asian medicine which caused the loss of ΔΨm and the accumulation of apoptotic bodies, indicating the induction of apoptosis [90]. A mitochondrial role of *Phyllanthus amarus* was also observed in HeLa, and CaSki cell lines. In these cells, the lignan-enriched fraction (LEF) triggered mitochondrial apoptosis mediated by the activation of p53, which induced an increase in Bax and a decrease in Bcl2. LEF also generated the production of ROS, which induced DNA damage and a decrease in ΔΨm. In addition, E6 decreased with treatment with LEF, suggesting that this product contains compounds with an affinity for this oncoprotein. Supporting the latter finding, the principal lignan of *Phyllanthus amarus*, extracted from the chloroform phase, showed an irreversible affinity for E6 according to an in silico analysis [91], which indicated that the inhibition of E6 is crucial for allowing apoptosis in cervical cancer cells. On the other hand, a lipid derived from *Pinellia pedatisecta* decreases the mRNA expression of E6 in HR-HPV-positive cervical cancer cell lines such as CaSki and HeLa cells, which was associated with an increase in both messenger RNA (mRNA) and protein levels of p53, Bax, and caspase-3, inducing mitochondrial apoptosis [92]. BCDD also downregulates the E6 mRNA of HR-HPV-18 from HeLa cells, inducing intrinsic apoptosis by restoring p53 and inducing the release of cyt c [81]. In other models of cancer, such as HNSCC HPV(+) or squamous cell carcinoma (SCC), it has been demonstrated that the mitochondria can be targeted to potentially reduce or eliminate these cancers. For instance, it has been found that in HNSCC HPV(+) cells, fenretinide (a retinoid derivative and an inducer of endoplasmic reticulum stress) induced the expression of the pro-apoptotic protein NOXA and led to mitochondrial apoptosis [93]. Moreover, in SCC, specifically in HPV-immortalized/v-Ha-Ras tumorigenic keratinocytes, the synthetic retinoid N-(4-hydroxyphenyl)retinamide (4-HPR) induced the production of ROS, mitochondrial membrane permeability transition (MPT), and apoptosis [94]. Thus, the different compounds reviewed above induce events such as the production of ROS, the activation of proapoptotic proteins, the deactivation of antiapoptotic proteins, the loss of Δψm, the release of cyt c, the activation of caspases, and, in general, lead to the activation of the different mitochondrial apoptosis pathways. This makes these compounds potential cancer drugs that target the mitochondria. However, further investigations are needed, as most of the studies have been conducted in cell models, while animal models would strengthen the present evidence of activity and would attract new experimental efforts in order to assay them regarding HPV-related therapy. HPV-related cancers, such as HNSCC HPV(+), could be targets for new biotherapies such as mitochondrial transplantation, in which functional mitochondria are transplanted into tumors with mitochondrial dysfunctions, activating mitochondrial apoptosis and having an antitumoral effect [95]. In HNSCC HPV(+) cancers, HPV proteins such as E6 induce mitochondrial dysfunction and may also decrease the mitochondrial apoptosis associated with the release of cytochrome c due to p53 decrease [54]. Therefore, transplantation of functional mitochondria could increase the intrinsic mechanisms of apoptosis due to the presence of mitochondria with an apoptotic system under adequate conditions to induce cell death. Moreover, this therapy could work adequately in HPV-related cervical cancer, where the remaining p53 that is not degraded by E6 could be sufficient to induce mitochondrial apoptosis through the newly transplanted healthy mitochondria. However, this is something that has still to be studied and deserves further urgent exploration, as mitochondria transplants could be an efficacious therapy against HPV-related cancers.

## 6. Conclusions

HPV-related cancers are dependent on the mitochondria because the mitochondria regulate the metabolism, survival, growth, and apoptosis of cancer cells. In these cancers, HR-HPV proteins bind to and modify the functions of the mitochondria, contributing to cell transformation; however, these HPV proteins can be targeted to be sensitized for eliminating HPV-related cancer, since different compounds or drugs can bind them, inducing mitochondrial apoptosis. Indeed, a predominant mechanism of action of the different compounds under investigation consists of inducing mitochondrial apoptosis by reducing the levels of HPV oncoproteins, so potential treatments with compounds that are able to induce mitochondrial apoptosis in HPV-related cancers cells may directly target HPV proteins. Therefore, the appropriate use of these molecules and the design of appropriate protocols targeting the downregulation of HPV oncoproteins to specifically induce mitochondrial apoptosis in HPV-related cancers may improve cancer survival in HPV-related neoplasia. 

## Figures and Tables

**Table 1:** Treatments (compounds or preparations) evaluated for targeting the mitochondria in HPV-related cancers.

	Mitochondria Targeting Treatment	Mitochondrial Proteins Involved	Regulation of HPV Oncoproteins	Effects	Ref.
HeLa cells	Rotenone	↑Bax	Not studied	↑ROS, ↓ATP, cell cycle arrest, ↑caspase-3, resulting in mitochondrial apoptosis	[79]
HeLa cells	TPT-benzimidazolethiol	↑Bax mRNA Cyt c and Smac/DIABLO release	↓E6 oncoprotein	↑p53, ↑caspase-3 and -9, G0/G1 cell cycle arrest, resulting in mitochondrial apoptosis	[80]
HeLa cells	Cyano derivative of 11-keto-β-boswellic acid, BCDD	↑Drp1 ↑Bax ↓Bcl2 ↑Cyt c release	↓E6 mRNA	↑p53, resulting in mitochondrial apoptosis	[81]
SiHa and HeLa cells	Berberine	↓ ΔΨm	↓E6 and E7 oncoproteins	↑Caspase-3, resulting in mitochondrial apoptosis and ↓cell viability	[82]
HeLa cells	EGCG and TF	Cyt c release	Not studied	↑ROS, ↑caspase-3 and -9, resulting in mitochondrial apoptosis	[84]
HeLa cells	DMMP	↑PUMA, ↑NOXA, ↑Bax, ↑Bad, ↑Bim, ↓ ΔΨm	Not studied	Cell cycle arrest in G1, mitochondrial apoptosis	[85]
HeLa, SiHa cells and a xenograft mouse model	Curcumin	↑Caspase-3 and -9 ↑Bax Cyt c release	Not studied	Mitochondrial apoptosis	[86]
SiHa cells and a xenograft mouse model	Atovaquone	Inhibits Complex III, inhibits mitochondrial respiration	Not studied	↓Cell viability	[87]
Caski and HeLa cells	Staurosporine	↑ Cyt c release	↓E6 and E7 oncoproteins	↑p53, ↑caspase-3 and -9, resulting in mitochondrial apoptosis	[88]
CaSki cell lines	Juglone	↑Bax ↓Bcl2 ↑ Cyt c release	Not studied	Cell cycle arrest in G2/M, ↑caspase-3, resulting in mitochondrial apoptosis	[89]
ME-180 and SiHa cell lines	Chloroform extract of *Rasagenthi mezhugu*	↓ ΔΨm	Not studied	Mitochondrial apoptosis	[90]
HeLa, and CaSki cell lines	LEF of *Phyllanthus amarus*	↑Bax ↓Bcl2 ↓ ΔΨm	Not studied	↑ROS, ↓E6, ↑p53, resulting in mitochondrial apoptosis	[91]
HeLa and CaSki cells	Lipid derived from *Pinellia pedatisecta*	↑ Bax	↓E6 mRNA	↑p53, ↑caspase-3, resulting in mitochondrial apoptosis	[92]
HNSCC HPV(+) cells	Fenretinide	↑NOXA	Not studied	Mitochondrial apoptosis	[93]
HNSCC HPV(+) cells	4-HPR	↑Caspase-3	Not studied	↑ROS, MTP, resulting in mitochondrial apoptosis	[94]

Abbreviations: Bax, Bcl-2-associated X protein; ROS, reactive oxygen species; ATP, adenosine triphosphate; TPT, triphenyl tin; Smac, second mitochondria derived activator of caspase; DIABLO, direct IAP binding protein with low pI; Drp1, dynamin-related protein 1; Bax, Bcl-2-associated X protein; Bcl2, B cell lymphoma; ΔΨm, mitochondrial membrane potential; LEF, lignan enriched fraction; EGCG, (−)-epigallocatechin gallate; TF, theaflavins; DMMP, 4-(3′,3′-dimethylallyloxy)-5-methyl-6-methoxy-phthalide; PUMA, p53 upregulated modulator of apoptosis; 4-HPR, N-(4-hydroxyphenyl)retinamide; MTP, mitochondrial membrane permeability transition; mRNA, messenger RNA; BCDD, butyl 2-cyano-3, 11-dioxours-1,12-dien-24-oate; cyt c, cytochrome c; HNSCC, head and neck squamous cell carcinoma. ↑: increase, ↓: decrease.

## Data Availability

Not applicable.

## Abbreviations

Apaf1: apoptotic protease activating factor-1; ATM/ATR, ataxia–telangiectasia mutated/RAD3-related; Bak, Bcl-2 homologous antagonist/killer; Bax, Bcl-2-associated X protein; BCDD, butyl 2-cyano-3, 11-dioxours-1,12-dien-24-oate; BCDD, butyl 2-cyano-3, 11-dioxours-1,12-dien-24-oate; Bcl2, B cell lymphoma; BS-IV, Buddlejasaponin IV; CAD, caspase-activated DNase; CAIX, carbonic anhydrase IX; CAT, catalase; cyt c, cytochrome c; DDR, DNA damage response; DHA, docosahexaenoic acid; DIABLO, direct IAP binding protein with low pI; DMMP, 4-(3′,3′-dimethylallyloxy)-5-methyl-6-methoxy-phthalide; DNA, deoxyribonucleic acid; Drp1, dynamin-related protein 1; E, early; EGCG, epigallocatechin gallate; ERK, extracellular signal-regulated kinases; ETS, electron transport system; FAO. fatty acid oxidation; FITC, fluorescein isothiocyanate; GPx, glutathione peroxidase; GSH, glutathione; H_2_O_2_, hydrogen peroxide; HAX-1, hematopoietic cell-specific protein 1-related protein X-1; HDAC, histone deacetylase; HIF 1α, hypoxia-inducible factor 1α; HNSCC, head and neck squamous cell carcinoma; 4-HPR, N-(4-hydroxyphenyl) retinamide; HR-HPV, high-risk human papillomavirus; hTERT, human telomerase reverse transcriptase; JNK, c-Jun N-terminal kinases; L, late; LCR, long control region; LEF, lignan-enriched fraction; MOMP, mitochondrial outer membrane permeabilization; MPT, membrane permeability transition; mRNA, messenger RNA; mROS, mitochondrial ROS; mtDNA, mitochondrial DNA; NADH, nicotinamide adenine dinucleotide; NFκB, nuclear factor kappa light-chain-enhancer of activated B cells; Nrf2, nuclear factor erythroid 2-related factor 2; O_2_^•−^, superoxide anion; ^•^OH, hydroxyl radical; OS, oxidative stress; OXPHOS, oxidative phosphorylation; PARP, poly-(ADP-ribose) polymerase; PDH, pyruvate dehydrogenase; PDK1, 3-phosphoinositide-dependent protein kinase 1; pRB, retinoblastoma tumor suppressor; PUMA, p53 upregulated modulator of apoptosis; ROS, reactive oxygen species; SCC, squamous cell carcinoma; Smac, second mitochondria derived activator of caspase; SOD, superoxidase dismutase; SP1, specificity protein 1; TCA, tricarboxylic acid cycle; TF, theaflavins; TFIID, transcription factor IID; TFs, transcription factors; TNF, tumor necrosis factor; TPT, triphenyl tin; UPS, ubiquitin–proteasome system; UVB, ultraviolet B; VEGF, vascular endothelial growth factor; WHO, World Health Organization; Δψm, mitochondrial membrane potential.

## References

[1] Sung H., Ferlay J., Siegel R.L., Laversanne M., Soerjomataram I., Jemal A., Bray F. (2021). Global Cancer Statistics 2020: GLOBOCAN Estimates of Incidence and Mortality Worldwide for 36 Cancers in 185 Countries. CA Cancer J. Clin..

[2] Walboomers J.M., Jacobs M.V., Manos M.M., Bosch F.X., Kummer J.A., Shah K.V., Snijders P.J., Peto J., Meijer C.J., Muñoz N. (1999). Human Papillomavirus Is a Necessary Cause of Invasive Cervical Cancer Worldwide. J. Pathol..

[3] Bray F., Ferlay J., Soerjomataram I., Siegel R.L., Torre L.A., Jemal A. (2018). Global Cancer Statistics 2018: GLOBOCAN Estimates of Incidence and Mortality Worldwide for 36 Cancers in 185 Countries. CA Cancer J. Clin..

[4] Egawa N., Doorbar J. (2017). The Low-Risk Papillomaviruses. Virus Res..

[5] Burd E.M. (2003). Human Papillomavirus and Cervical Cancer. Clin. Microbiol. Rev..

[6] de Villiers E.-M. (2013). Cross-Roads in the Classification of Papillomaviruses. Virology.

[7] IARC Working Group on the Evaluation of Carcinogenic Risks to Humans (2007). Human Papillomavirus (HPV) Infection.

[8] Doorbar J., Quint W., Banks L., Bravo I.G., Stoler M., Broker T.R., Stanley M.A. (2012). The Biology and Life-Cycle of Human Papillomaviruses. Vaccine.

[9] Zheng Z.-M., Baker C.C. (2006). Papillomavirus genome structure, expression, and post-transcriptional regulation. Front. Biosci..

[10] Dasgupta J., Bienkowska-Haba M., Ortega M.E., Patel H.D., Bodevin S., Spillmann D., Bishop B., Sapp M., Chen X.S. (2011). Structural Basis of Oligosaccharide Receptor Recognition by Human Papillomavirus. J. Biol. Chem..

[11] Gravitt P.E., Winer R.L. (2017). Natural History of HPV Infection across the Lifespan: Role of Viral Latency. Viruses.

[12] Aksoy P., Gottschalk E.Y., Meneses P.I. (2017). HPV Entry into Cells. Mutat. Res. Rev. Mutat. Res..

[13] Ribeiro A.L., Caodaglio A.S., Sichero L. (2018). Regulation of HPV Transcription. Clinics.

[14] Zhang B., Chen W., Roman A. (2006). The E7 Proteins of Low- and High-Risk Human Papillomaviruses Share the Ability to Target the PRB Family Member P130 for Degradation. Proc. Natl. Acad. Sci. USA.

[15] Tomaić V. (2016). Functional Roles of E6 and E7 Oncoproteins in HPV-Induced Malignancies at Diverse Anatomical Sites. Cancers.

[16] Venuti A., Paolini F., Nasir L., Corteggio A., Roperto S., Campo M.S., Borzacchiello G. (2011). Papillomavirus E5: The Smallest Oncoprotein with Many Functions. Mol. Cancer.

[17] Martínez-Ramírez I., Carrillo-García A., Contreras-Paredes A., Ortiz-Sánchez E., Cruz-Gregorio A., Lizano M. (2018). Regulation of Cellular Metabolism by High-Risk Human Papillomaviruses. Int. J. Mol. Sci..

[18] Cruz-Gregorio A., Aranda-Rivera A.K., Pedraza-Chaverri J. (2020). Human Papillomavirus-Related Cancers and Mitochondria. Virus Res..

[19] Sun L., Zhang G., Lei T., Huang C., Song T., Si L. (2008). Two Different HPV-11E6 Fusion Proteins Trap P53 in the Cytoplasm and Induce Apoptosis. Cancer Biol. Ther..

[20] Scheffner M., Werness B.A., Huibregtse J.M., Levine A.J., Howley P.M. (1990). The E6 Oncoprotein Encoded by Human Papillomavirus Types 16 and 18 Promotes the Degradation of P53. Cell.

[21] Leverrier S., Bergamaschi D., Ghali L., Ola A., Warnes G., Akgül B., Blight K., García-Escudero R., Penna A., Eddaoudi A. (2007). Role of HPV E6 Proteins in Preventing UVB-Induced Release of pro-Apoptotic Factors from the Mitochondria. Apoptosis.

[22] Vyas S., Zaganjor E., Haigis M.C. (2016). Mitochondria and Cancer. Cell.

[23] Wallace D.C. (2012). Mitochondria and Cancer. Nat. Rev. Cancer.

[24] Valko M., Leibfritz D., Moncol J., Cronin M.T.D., Mazur M., Telser J. (2007). Free Radicals and Antioxidants in Normal Physiological Functions and Human Disease. Int. J. Biochem. Cell Biol..

[25] Cruz-Gregorio A., Martínez-Ramírez I., Pedraza-Chaverri J., Lizano M. (2019). Reprogramming of Energy Metabolism in Response to Radiotherapy in Head and Neck Squamous Cell Carcinoma. Cancers.

[26] Dinkova-Kostova A.T., Holtzclaw W.D., Cole R.N., Itoh K., Wakabayashi N., Katoh Y., Yamamoto M., Talalay P. (2002). Direct Evidence That Sulfhydryl Groups of Keap1 Are the Sensors Regulating Induction of Phase 2 Enzymes That Protect against Carcinogens and Oxidants. Proc. Natl. Acad. Sci. USA.

[27] Putker M., Vos H.R., van Dorenmalen K., de Ruiter H., Duran A.G., Snel B., Burgering B.M.T., Vermeulen M., Dansen T.B. (2015). Evolutionary Acquisition of Cysteines Determines FOXO Paralog-Specific Redox Signaling. Antioxid. Redox Signal..

[28] Klotz L.-O., Sánchez-Ramos C., Prieto-Arroyo I., Urbánek P., Steinbrenner H., Monsalve M. (2015). Redox Regulation of FoxO Transcription Factors. Redox Biol..

[29] Holmström K.M., Finkel T. (2014). Cellular Mechanisms and Physiological Consequences of Redox-Dependent Signalling. Nat. Rev. Mol. Cell Biol..

[30] Cadenas E. (1997). Basic Mechanisms of Antioxidant Activity. Biofactors.

[31] Kryston T.B., Georgiev A.B., Pissis P., Georgakilas A.G. (2011). Role of Oxidative Stress and DNA Damage in Human Carcinogenesis. Mutat. Res..

[32] Valko M., Rhodes C.J., Moncol J., Izakovic M., Mazur M. (2006). Free Radicals, Metals and Antioxidants in Oxidative Stress-Induced Cancer. Chem. Biol. Interact..

[33] Cruz-Gregorio A., Manzo-Merino J., Lizano M. (2018). Cellular Redox, Cancer and Human Papillomavirus. Virus Res..

[34] Murphy M.P. (2009). How Mitochondria Produce Reactive Oxygen Species. Biochem. J..

[35] Liou G.-Y., Storz P. (2010). Reactive Oxygen Species in Cancer. Free Radic. Res..

[36] Craig A., Scott M., Burch L., Smith G., Ball K., Hupp T. (2003). Allosteric Effects Mediate CHK2 Phosphorylation of the P53 Transactivation Domain. EMBO Rep..

[37] Zilfou J.T., Lowe S.W. (2009). Tumor Suppressive Functions of P53. Cold Spring Harb. Perspect. Biol..

[38] Colotta F., Allavena P., Sica A., Garlanda C., Mantovani A. (2009). Cancer-Related Inflammation, the Seventh Hallmark of Cancer: Links to Genetic Instability. Carcinogenesis.

[39] Willis S.N., Adams J.M. (2005). Life in the Balance: How BH3-Only Proteins Induce Apoptosis. Curr. Opin. Cell Biol..

[40] Jiang X., Wang X. (2004). Cytochrome C-Mediated Apoptosis. Annu. Rev. Biochem..

[41] Yoshida H., Kong Y.Y., Yoshida R., Elia A.J., Hakem A., Hakem R., Penninger J.M., Mak T.W. (1998). Apaf1 Is Required for Mitochondrial Pathways of Apoptosis and Brain Development. Cell.

[42] Jiang X., Wang X. (2000). Cytochrome c Promotes Caspase-9 Activation by Inducing Nucleotide Binding to Apaf-1. J. Biol. Chem..

[43] Brentnall M., Rodriguez-Menocal L., De Guevara R.L., Cepero E., Boise L.H. (2013). Caspase-9, Caspase-3 and Caspase-7 Have Distinct Roles during Intrinsic Apoptosis. BMC Cell Biol..

[44] Nagata S., Nagase H., Kawane K., Mukae N., Fukuyama H. (2003). Degradation of Chromosomal DNA during Apoptosis. Cell Death Differ..

[45] Bratton D.L., Fadok V.A., Richter D.A., Kailey J.M., Guthrie L.A., Henson P.M. (1997). Appearance of Phosphatidylserine on Apoptotic Cells Requires Calcium-Mediated Nonspecific Flip-Flop and Is Enhanced by Loss of the Aminophospholipid Translocase. J. Biol. Chem..

[46] McBride A.A. (2013). The Papillomavirus E2 Proteins. Virology.

[47] Lai D., Tan C.L., Gunaratne J., Quek L.S., Nei W., Thierry F., Bellanger S. (2013). Localization of HPV-18 E2 at Mitochondrial Membranes Induces ROS Release and Modulates Host Cell Metabolism. PLoS ONE.

[48] Cruz-Gregorio A., Manzo-Merino J., Gonzaléz-García M.C., Pedraza-Chaverri J., Medina-Campos O.N., Valverde M., Rojas E., Rodríguez-Sastre M.A., García-Cuellar C.M., Lizano M. (2018). Human Papillomavirus Types 16 and 18 Early-Expressed Proteins Differentially Modulate the Cellular Redox State and DNA Damage. Int. J. Biol. Sci..

[49] Gao L., Gu P., Zhao W., Ding W., Zhao X., Guo S., Zhong T. (2013). The Role of Globular Heads of the C1q Receptor in HPV 16 E2-Induced Human Cervical Squamous Carcinoma Cell Apoptosis Is Associated with P38 MAPK/JNK Activation. J. Transl. Med..

[50] Chen Z., Su Y., Zhang H., Gu P., Gao L. (2014). The Role of the Globular Heads of the C1q Receptor in HPV-16 E2-Induced Human Cervical Squamous Carcinoma Cell Apoptosis via a Mitochondria-Dependent Pathway. J. Transl. Med..

[51] Gong H., Wang P., Yu M., Zhu Y., Teng L., Su Y. (2021). The Role of the Hematopoietic Cell-Specific Protein 1-Associated Protein X-1 in Human Papillomavirus 16 E2-Induced Human Cervical Squamous Carcinoma Cell Apoptosis via a Mitochondria-Dependent Pathway. Gynecol. Obs. Investig..

[52] Doorbar J. (2013). The E4 Protein; Structure, Function and Patterns of Expression. Virology.

[53] Raj K., Berguerand S., Southern S., Doorbar J., Beard P. (2004). E1 Empty Set E4 Protein of Human Papillomavirus Type 16 Associates with Mitochondria. J. Virol..

[54] Cruz-Gregorio A., Aranda-Rivera A.K., Aparicio-Trejo O.E., Coronado-Martínez I., Pedraza-Chaverri J., Lizano M. (2019). E6 Oncoproteins from High-Risk Human Papillomavirus Induce Mitochondrial Metabolism in a Head and Neck Squamous Cell Carcinoma Model. Biomolecules.

[55] Pim D., Massimi P., Banks L. (1997). Alternatively Spliced HPV-18 E6* Protein Inhibits E6 Mediated Degradation of P53 and Suppresses Transformed Cell Growth. Oncogene.

[56] Evans W., Filippova M., Filippov V., Bashkirova S., Zhang G., Reeves M.E., Duerksen-Hughes P. (2016). Overexpression of HPV16 E6* Alters β-Integrin and Mitochondrial Dysfunction Pathways in Cervical Cancer Cells. Cancer Genom. Proteom..

[57] Tamura R.E., de Vasconcellos J.F., Sarkar D., Libermann T.A., Fisher P.B., Zerbini L.F. (2012). GADD45 Proteins: Central Players in Tumorigenesis. Curr. Mol. Med..

[58] Cho C.W., Poo H., Cho Y.S., Cho M.C., Lee K.A., Lee S.J., Park S.N., Kim I.K., Jung Y.K., Choe Y.K. (2002). HPV E6 Antisense Induces Apoptosis in CaSki Cells via Suppression of E6 Splicing. Exp. Mol. Med..

[59] Vogt M., Butz K., Dymalla S., Semzow J., Hoppe-Seyler F. (2006). Inhibition of Bax Activity Is Crucial for the Antiapoptotic Function of the Human Papillomavirus E6 Oncoprotein. Oncogene.

[60] Cherry J.J., Rietz A., Malinkevich A., Liu Y., Xie M., Bartolowits M., Davisson V.J., Baleja J.D., Androphy E.J. (2013). Structure Based Identification and Characterization of Flavonoids That Disrupt Human Papillomavirus-16 E6 Function. PLoS ONE.

[61] Clemente-Soto A.F., Salas-Vidal E., Milan-Pacheco C., Sánchez-Carranza J.N., Peralta-Zaragoza O., González-Maya L. (2019). Quercetin Induces G2 Phase Arrest and Apoptosis with the Activation of P53 in an E6 Expression-Independent Manner in HPV-Positive Human Cervical Cancer-Derived Cells. Mol. Med. Rep..

[62] Zhou Y., Wei Y., Zhu J., Wang Q., Bao L., Ma Y., Chen Y., Feng D., Zhang A., Sun J. (2011). GRIM-19 Disrupts E6/E6AP Complex to Rescue P53 and Induce Apoptosis in Cervical Cancers. PLoS ONE.

[63] Singh M., Singh N. (2008). Induction of Apoptosis by Hydrogen Peroxide in HPV 16 Positive Human Cervical Cancer Cells: Involvement of Mitochondrial Pathway. Mol. Cell Biochem..

[64] Boyer S.N., Wazer D.E., Band V. (1996). E7 Protein of Human Papilloma Virus-16 Induces Degradation of Retinoblastoma Protein through the Ubiquitin-Proteasome Pathway. Cancer Res..

[65] Shim J.-H., Kim K.-H., Cho Y.-S., Choi H.-S., Song E.Y., Myung P.-K., Kang J.S., Suh S.-K., Park S.N., Yoon D.-Y. (2008). Protective Effect of Oxidative Stress in HaCaT Keratinocytes Expressing E7 Oncogene. Amino Acids.

[66] Finzer P., Krueger A., Stöhr M., Brenner D., Soto U., Kuntzen C., Krammer P.H., Rösl F. (2004). HDAC Inhibitors Trigger Apoptosis in HPV-Positive Cells by Inducing the E2F-P73 Pathway. Oncogene.

[67] Lee S., Kim H., Kang J.-W., Kim J.-H., Lee D.H., Kim M.-S., Yang Y., Woo E.-R., Kim Y.M., Hong J. (2011). The Biflavonoid Amentoflavone Induces Apoptosis via Suppressing E7 Expression, Cell Cycle Arrest at Sub-G₁ Phase, and Mitochondria-Emanated Intrinsic Pathways in Human Cervical Cancer Cells. J. Med. Food.

[68] Gao L.-J., Gu P.-Q., Fan W.-M., Liu Z., Qiu F., Peng Y.-Z., Guo X.-R. (2011). The Role of GC1qR in Regulating Survival of Human Papillomavirus 16 Oncogene-Transfected Cervical Cancer Cells. Int. J. Oncol..

[69] Hwang Y.S., Chung W.-Y., Kim J., Park H.-J., Kim E.-C., Park K.-K. (2011). Buddlejasaponin IV Induces Cell Cycle Arrest at G2/M Phase and Apoptosis in Immortalized Human Oral Keratinocytes. Phytother Res..

[70] Jing K., Shin S., Jeong S., Kim S., Song K.-S., Park J.-H., Heo J.-Y., Seo K.-S., Park S.-K., Kweon G.-R. (2014). Docosahexaenoic Acid Induces the Degradation of HPV E6/E7 Oncoproteins by Activating the Ubiquitin-Proteasome System. Cell Death Dis..

[71] Kabsch K., Alonso A. (2002). The Human Papillomavirus Type 16 (HPV-16) E5 Protein Sensitizes Human Keratinocytes to Apoptosis Induced by Osmotic Stress. Oncogene.

[72] Brown J., Higo H., McKalip A., Herman B. (1997). Human Papillomavirus (HPV) 16 E6 Sensitizes Cells to Atractyloside-Induced Apoptosis: Role of P53, ICE-like Proteases and the Mitochondrial Permeability Transition. J. Cell. Biochem..

[73] Vikhanskaya F., Falugi C., Valente P., Russo P. (2002). Human Papillomavirus Type 16 E6-Enhanced Susceptibility to Apoptosis Induced by TNF in A2780 Human Ovarian Cancer Cell Line. Int. J. Cancer.

[74] Thomas R.J., Oleinik N., Panneer Selvam S., Vaena S.G., Dany M., Nganga R.N., Depalma R., Baron K.D., Kim J., Szulc Z.M. (2017). HPV/E7 Induces Chemotherapy-Mediated Tumor Suppression by Ceramide-Dependent Mitophagy. EMBO Mol. Med..

[75] Sun W., Qin X., Zhou J., Xu M., Lyu Z., Li X., Zhang K., Dai M., Li N., Hang D. (2020). Mitochondrial DNA copy number in cervical exfoliated cells and risk of cervical cancer among HPV-positive women. BMC Womens Health..

[76] Guo Y., Meng X., Ma J., Zheng Y., Wang Q., Wang Y., Shang H. (2014). Human Papillomavirus 16 E6 Contributes HIF-1α Induced Warburg Effect by Attenuating the VHL-HIF-1α Interaction. Int. J. Mol. Sci..

[77] Fan R., Hou W.-J., Zhao Y.-J., Liu S.-L., Qiu X.-S., Wang E.-H., Wu G.-P. (2016). Overexpression of HPV16 E6/E7 Mediated HIF-1α Upregulation of GLUT1 Expression in Lung Cancer Cells. Tumour Biol..

[78] Zhai K., Chang L., Zhang Q., Liu B., Wu Y. (2011). Mitochondrial C150T Polymorphism Increases the Risk of Cervical Cancer and HPV Infection. Mitochondrion.

[79] Agarwal N.R., Maurya N., Pawar J.S., Ghosh I. (2016). A Combined Approach against Tumorigenesis Using Glucose Deprivation and Mitochondrial Complex 1 Inhibition by Rotenone. Cell Biol. Int..

[80] Höti N., Ma J., Tabassum S., Wang Y., Wu M. (2003). Triphenyl Tin Benzimidazolethiol, a Novel Antitumor Agent, Induces Mitochondrial-Mediated Apoptosis in Human Cervical Cancer Cells via Suppression of HPV-18 Encoded E6. J. Biochem..

[81] Khan S., Chib R., Shah B.A., Wani Z.A., Dhar N., Mondhe D.M., Lattoo S., Jain S.K., Taneja S.C., Singh J. (2011). A Cyano Analogue of Boswellic Acid Induces Crosstalk between P53/PUMA/Bax and Telomerase That Stages the Human Papillomavirus Type 18 Positive HeLa Cells to Apoptotic Death. Eur. J. Pharmacol..

[82] Marasco D., Vicidomini C., Krupa P., Cioffi F., Huy P.D.Q., Li M.S., Florio D., Broersen K., De Pandis M.F., Roviello G.N. (2021). Plant Isoquinoline Alkaloids as Potential Neurodrugs: A Comparative Study of the Effects of Benzo[c]Phenanthridine and Berberine-Based Compounds on β-Amyloid Aggregation. Chem. Biol. Interact..

[83] Mahata S., Bharti A.C., Shukla S., Tyagi A., Husain S.A., Das B.C. (2011). Berberine Modulates AP-1 Activity to Suppress HPV Transcription and Downstream Signaling to Induce Growth Arrest and Apoptosis in Cervical Cancer Cells. Mol. Cancer.

[84] Singh M., Singh R., Bhui K., Tyagi S., Mahmood Z., Shukla Y. (2011). Tea Polyphenols Induce Apoptosis Through Mitochondrial Pathway and by Inhibiting Nuclear Factor-ΚB and Akt Activation in Human Cervical Cancer Cells. Oncol. Res..

[85] Chen C., Hu S.-Y., Luo D.-Q., Zhu S.-Y., Zhou C.-Q. (2013). Potential Antitumor Agent from the Endophytic Fungus Pestalotiopsis Photiniae Induces Apoptosis via the Mitochondrial Pathway in HeLa Cells. Oncol. Rep..

[86] Singh M., Singh N. (2009). Molecular Mechanism of Curcumin Induced Cytotoxicity in Human Cervical Carcinoma Cells. Mol. Cell Biochem..

[87] Tian S., Chen H., Tan W. (2018). Targeting Mitochondrial Respiration as a Therapeutic Strategy for Cervical Cancer. Biochem. Biophys. Res. Commun..

[88] Bernard B., Prétet J.-L., Charlot J.-F., Mougin C. (2003). Human Papillomaviruses Type 16+ and 18+ Cervical Carcinoma Cells Are Sensitive to Staurosporine-Mediated Apoptosis. Biol. Cell.

[89] Zhao X., Song X., Zhao J., Zhu W., Hou J., Wang Y., Zhang W. (2019). Juglone Inhibits Proliferation of HPV-Positive Cervical Cancer Cells Specifically. Biol. Pharm. Bull..

[90] Riyasdeen A., Periasamy V.S., Paul P., Alshatwi A.A., Akbarsha M.A. (2012). Chloroform Extract of Rasagenthi Mezhugu, a Siddha Formulation, as an Evidence-Based Complementary and Alternative Medicine for HPV-Positive Cervical Cancers. Evid. Based Complement. Altern. Med..

[91] Paul S., Patra D., Kundu R. (2019). Lignan Enriched Fraction (LRF) of Phyllanthus Amarus Promotes Apoptotic Cell Death in Human Cervical Cancer Cells in Vitro. Sci. Rep..

[92] Li G.-L., Jiang W., Xia Q., Chen S.-H., Ge X.-R., Gui S.-Q., Xu C.-J. (2010). HPV E6 Down-Regulation and Apoptosis Induction of Human Cervical Cancer Cells by a Novel Lipid-Soluble Extract (PE) from Pinellia Pedatisecta Schott in Vitro. J. Ethnopharmacol..

[93] Britt E.L., Raman S., Leek K., Sheehy C.H., Kim S.W., Harada H. (2019). Combination of Fenretinide and ABT-263 Induces Apoptosis through NOXA for Head and Neck Squamous Cell Carcinoma Treatment. PLoS ONE.

[94] Bruno S., Tenca C., Saverino D., Ciccone E., Grossi C.E. (2002). Apoptosis of Squamous Cells at Different Stages of Carcinogenesis Following 4-HPR Treatment. Carcinogenesis.

[95] Chang J.-C., Chang H.-S., Wu Y.-C., Cheng W.-L., Lin T.-T., Chang H.-J., Kuo S.-J., Chen S.-T., Liu C.-S. (2019). Mitochondrial Transplantation Regulates Antitumour Activity, Chemoresistance and Mitochondrial Dynamics in Breast Cancer. J. Exp. Clin. Cancer Res..

